# Paxlovid plus glucocorticoids treatment in severe Omicron infected patients with hypoxaemia: A prospective multiple-center cohort study

**DOI:** 10.1371/journal.pone.0328929

**Published:** 2025-09-26

**Authors:** Yu-Ji Wang, Nan Su, Jun-Hong Jiang, Guo-Peng Xu, Ran Wang, Da-Xiong Zeng, Jiao Zhu

**Affiliations:** 1 Department of pulmonary and critical care medicine, The Fourth Affiliated Hospital of Soochow University, Suzhou Dushu Lake Hospital, Suzhou, People's Republic of China; 2 Medical Center of Soochow University, Suzhou, People's Republic of China; 3 Department of Pulmonary and Critical Care Medicine, First Affiliated Hospital of Soochow University, Suzhou, People's Republic of China; 4 Department of Pulmonary and Critical Care, The Affiliated Suzhou Hospital of Nanjing Medical University, Suzhou Municipal Hospital, Suzhou, People's Republic of China; 5 Gusu School, Nanjing Medical University, Suzhou, People's Republic of China; 6 Department of Pulmonary and Critical Care, The Affiliated Hospital of Anhui Medical University, Hefei, People's Republic of China; 7 Department of obstetrics and gynecology, First Affiliated Hospital of Soochow University, Suzhou, People's Republic of China; Environmental Research Center (CRE), ALGERIA

## Abstract

**Background:**

Administration of Paxlovid in early stage has been proved to reduce the risk of hospitalization or death by 89% in mild to moderate COVID-19 patients with high-risk. There were few evidences of Paxlovid in severe COVID-19 patients. RECOVERY study has previously shown that the use of glucocorticoids reduces the risk of death in hospitalized COVID-19 patients requiring oxygen or ventilatory support. The efficacy of Paxlovid plus glucocorticoids in COVID-19 patients with hypoxaemia is unclear.

**Methods:**

In this multiple-centers prospective study, we collected the data of hospitalized adult Omicron infected subjects with hypoxaemia at 4 hospitals, who were treated with glucocorticoids or Paxlovid plus glucocorticoid. We compared the efficacy of Paxlovid plus glucocorticoids (P + GCS group) vs. glucocorticoids (GCS group). A 28-day composite outcome of disease progression was evaluated.

**Results:**

Totally 266 Omicron infected patients with hypoxaemia were enrolled in this study. There was no difference in most of the baseline characteristics in two groups, including ages, sex and underlying diseases. The 28-day composite outcome in severe patients of P + GCS group was significantly lower than that of GCS group (16.9% vs. 33.8%, P = 0.013). The viral shedding time was shorter in P + GCS group than that in G group (6 days vs. 8 days, P = 0.015). The hospitalized time in severe patients of P + GCS group was significantly shorter than that of GCS group (15 days vs. 17 days, P = 0.0008). Cox analysis showed the benefit of P + GCS in sub-group of MODS, CRP ≥ 36 mg/L, d-dimer ≥1 µg/L, creatinine ≥ 90µmol/L).

**Conclusions:**

Our study demonstrated the benefit of Paxlovid plus glucocorticoid administration in hospitalized Omicron infection patients with hypoxaemia. These results, as least partly, supported direct evidence about the necessity of antiviral treatment in severe COVID-19 patients with hypoxaemia.

## Introduction

As a highly contagious infectious disease, Coronavirus disease 2019 (COVID-19) remains a serious threat to global health, especially in older subjects or with underlying diseases [1]. Paxlovid, the combination of nirmatrelvir and ritonavir, selectively and reversibly prevented SARS-CoV-2 virus from making any functional proteins to replicate [2]. Previous EPIC-HR study has proved that Paxlovid reduced hospitalization and 28-day mortality rates by 89.1% and 88.9% respectively in ambulatory and unvaccinated COVID-19 patients [3]. Paxlovid also reduced risk of disease progression by 46% in COVID-19 patients with at least 1 comorbidity or condition associated with high risk for severe disease [4–6]. In these studies, Paxlovid was administrated in mild to moderate COVID-19 patients at early stage after disease onset. There is little evidence about Paxlovid in COVID-19 patients with hypoxaemia or severe subjects.

For COVID-19 patients with hypoxaemia and subjects with severe or critical ill, glucocorticoids may inhibit cytokines storm and modulate inflammation-mediated lung injury, which reduces progression to respiratory failure and death [7]. RECOVERY study and other reports has previously shown that the use of low-dose corticosteroids reduces the risk of death in hospitalized COVID-19 patients requiring oxygen or ventilatory support [8–10]. Subsequent findings confirmed that additional immunosuppression with an IL-6 receptor blocker or a Janus kinase (JAK) inhibitor further reduces the risk of death in these patients [11,12]. These data identified the importance of anti-inflammation treatment in COVID-19 patients with hypoxaemia. However, multiple reports indicated that usage of corticosteroids could delay the viral clearance, which might result in virus rebound and secondary cytokines storm [13–15]. It is unclear whether additional extended anti-viral treatment was beneficial for these hypoxic patients or not.

A few studies focused on the combination treatment of Remdesivir and dexamethasone in COVID-19, but with different or even contradictory conclusions. Several studies indicated that remdesivir plus dexamethasone treatment is associated with significant reduction in mortality, length of hospitalization, and faster SARS-CoV-2 clearance [16,17]. However, other reports found no association with shorter hospitalization or lower in-hospital mortality [18,19]. Furthermore, few evidence was found about other antiviral drugs combination with glucocorticoids in COVID-19 patients.

In this prospective cohort study, we aimed to evaluate the clinical efficacy of Paxlovid plus glucocorticoids in hospitalized COVID-19 patients with hypoxaemia in four hospitals.

## Methods

### Study design

During the period from Dec 1, 2022 to May 30, 2023, we performed a prospective cohort study of hospitalized COVID-19 patients with hypoxaemia in four centers (the Fourth Affiliated Hospital of Soochow University, the First Affiliated Hospital of Anhui Medical University, the First Affiliated Hospital of Soochow University, the Affiliated Suzhou Hospital of Nanjing Medical University). All subjects were diagnosed and confirmed for SARS-CoV-2 infection by positive RT-PCR. All patients were treated by their attending physician without additional interferes according to the diseases severity and drugs accessibility. We only enrolled the subjects with hypoxaemia and treated by glucocorticoids or Paxlovid plus glucocorticoids, except for usual care (including antibiotics, oxygen therapy, symptomatic treatment etc). The exclusion criteria were: (1) younger than 18 years; (2) died in 48h after hospitalization; (3) received Paxlovid administration for less than 5 days; (4) received other antiviral agents except for Paxlovid; (5) received mechanical ventilation on admission; (6) patients with immunodeficiency or using immunosuppressive drugs; (7) pregnant or lactation period; (8) the clinical data and prognosis information were missed.

This study was approved by the institutional review board of the Fourth Affiliated Hospital of Soochow University (2023404). The written informed consent was waived as we only collected the clinical data from anonymized data without additional drugs or treatment, according to the policy for public health outbreak investigation of emerging infectious diseases issued by the National Health Commission of the People’s Republic of China.

### Data collection

We collected the demographic characteristics (age, sex, underlying diseases) and clinical data (diagnosis, treatment, changes of plasma parameters, blood gas oxygenation index) from electronic health records in four hospitals. We also recorded the severity of COVID-19 on admission, and the respiratory support approaches (nasal catheter, high-flow nasal oxygen or noninvasive ventilation). All data were independently reviewed and entered into the computer database by 2 physicians.

The primary endpoint was a 28-day composite outcomes of disease progression, including all-cause death, intensive care unit admission, noninvasive or invasive mechanical ventilation, whichever came first. The secondary endpoint was viral clearance and hospitalization time.

Severe subjects were defined having one or more of the followings: (1) respiratory rate ≥30, (2) lung infiltrates >50%, (3) oxygen saturation ≤ 93%, (4) PaO_2_/FiO_2_ ≤ 300 mmHg. Critical subjects were defined when he/she had one or more of the followings: (1) shock requiring vasopressor support, (2) required intensive care unit management because of a combination of other organ failures, (3) respiratory failure requiring ventilator treatment.

### Statistical analysis

Statistical analysis was performed using SPSS (version 15.0). The normal distribution data of continuous variables were presented as average and standard deviation. The skew distribution data of continuous variables were presented as median and quartile. Categorical variables were presented as n (%) respectively. X^2^ test or Mann-Whitney U test was used to compare differences between early group and delayed group. Cox analysis were used for confirming the independent risk factors. Survival curves were plotted using the Kaplan-Meier method by the log-rank test. The level of significance was two-tailed 0.05 for statistical tests.

## Results

### Baseline characteristics of two groups

We consecutively collected data of 266 confirmed COVID-19 patients from 4 centers in Suzhou City and Hefei City. All the baseline characteristics are shown in Table 1. There was no difference between two groups in age, sex, BMI and underlying diseases. There were more severe patients in Paxlovid plus GCS group (65.7% vs. 42.5%, *P* = 0.001). The levels of CRP [42.50(14.86, 94.00) mg/L *vs* 18.00(8.53,65.25) mg/L, *P* = 0.049] and IL6 [15.65(7.16,46.80) pg/ml *vs* 4.94(0,12.83) pg/ml, *P* = 0.001] were significantly higher, but lymphocytes were lower in Paxlovid plus GCS group [0.79(0.44,1.24)×10^9^/L *vs* 0.95(0.66,1.41)×10^9^/L, *P* = 0.009]. The levels of hemoglobin were higher in Paxlovid plus GCS group than that in GCS group [129.50(115.00,142.50) g/L *vs* 123.00 (107.50,134.00) g/L, *P* = 0.022]. The levels of D-dimer, Lactic dehydrogenase procalcitonin and troponin were similar in two groups.

**Table 1:** Baseline characteristics of demography, blood parameters and treatment in Omicron infected patients in two groups.

	Paxlovid plus GCS (n = 99)	GCS (N = 167)	*P* value
**Age, yr**	74.41 ± 13.10	69.83 ± 15.69	0.027
**gendar**			0.08
male	64(64.6%)	92(55.1%)	
female	35(35.4%)	75(44.9%)	
**Body Mass Index**	23.318 ± 3.690	23.462 ± 3.665	0.78
**Vaccination**	81 (81.8)	148 (88.6)	0.12
**Severity(n,%)**			0.001
Moderate	34(34.3%)	96(57.5%)	
Severe	65(65.7%)	71(42.5%)	
**Comorbidity**			
Diabetes	21 (21.2%)	44 (26.3%)	0.214
Hypertension	54 (54.5%)	104 (62.3%)	0.133
Coronary heart disease	9 (9.1%)	15 (9.0%)	0.57
Cerebrovascular disease	6 (6.1%)	13 (7.8%)	0.065
Chronic pulmonary disease	14 (14.1%)	13 (7.8%)	0.075
Chronic kidney disease	6 (6.1%)	8 (4.8%)	0.427
Immune rheumatism	5 (5.1%)	2 (1.2%)	0.069
Tumor	18 (18.2%)	19 (11.4%)	0.087
**Lab findings**			
Leukocytes, 10^9^/L	6.39 (4.16,9.09)	6.04 (4.66,9.17)	0.696
Lymphocytes, 10^9^/L	0.79 (0.44,1.24)	0.95 (0.66,1.41)	0.009
Platelets, 10^12^/L	169.50 (130.25,238.75)	190.00 (151.00,246.00)	0.306
Hemoglobin, g/L	129.50 (115.00,142.50)	123.00 (107.50,134.00)	0.022
C reactive protein, mg/L	42.50 (14.86,94.00)	18.00 (8.53,65.25)	0.049
IL-6, pg/ml	15.65 (7.16,46.80)	4.94 (0,12.83)	0.001
Albumin, g/L	34.50 (28.50,37.60)	33.90 (29.90,37.80)	0.835
Creatinine, µmol/L	66.60 (57.15,88.45)	65.00 (51.60,93.80)	0.747
NT-proBNP, pg/ml	350.00 (219.35,894.85)	340.00 (77.56,1012.50)	0.327
D-dimer, mg/L	0.94 (0.50,1.70)	0.77 (0.39,1.67)	0.403
Lactic dehydrogenase, IU/L	216.65 (185.45,270.15)	210.00 (171.23,292.45)	0.659
Procalcitonin, ng/ml	0.08 (0.033,0.21)	0.05 (0.02,0.19)	0.466
Troponin, pg/ml	15.80 (9.57,26.41)	14.14 (7.02,25.20)	0.257
**Respiratory support, n (%)**			
None	11 (11.1%)	39 (23.5%)	0.013
Nasal catheter	58 (58.6%)	96 (57.8%)	0.86
Venturi mask	3(3.0%)	7 (4.2%)	0.63
high-flow nasal oxygen	14 (14.1%)	6 (3.6%)	0.001
Noninvasive ventilation	5 (5.1%)	3 (1.8%)	0.133
Invasive ventilation	8 (8.1%)	15 (8.7%)	0.8
**Treatment**			
Duration of Paxlovid therapy, days	6.12 ± 3.57	--	--
Duration of GCS therapy	9(5, 14)	11(7, 16)	0.148
Average dose (DXM, mg/d)	7.28 (4.73, 9.52)	7.76 (3.91, 9.68)	0.759
Antibiotics, n(%)	89 (89.9%)	146 (87.4%)	0.693

**GCS**: glucocorticoids; **DXM**: dexamethasone.

Categorical variables were presented as n (%) respectively. The normal distribution data of continuous variables were presented as average and standard deviation. The skew distribution data of continuous variables were presented as median and quartile.

More patients received high-flow nasal oxygen treatment in Paxlovid plus GCS group (14.1% *vs* 3.6%, *P* = 0.001). Less patients received no respiratory support in Paxlovid plus GCS group (11.1% *vs* 23.5%, *P* = 0.013). The ratio of patients received noninvasive ventilation or invasive ventilation treatment were similar. It was similar between two groups in duration and average dose of GCS treatment.

### Outcomes of Omicron infected patients after different treatment

**Fig 1:**
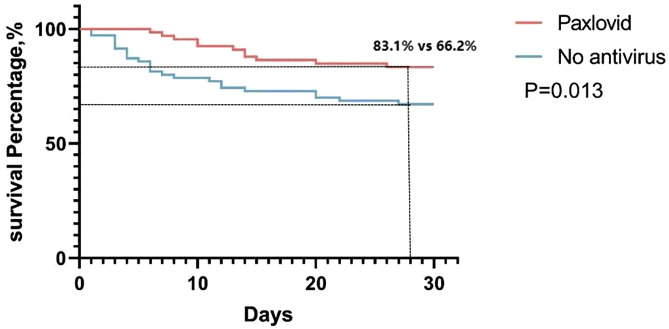
Kaplan-Meier analysis showed the 28-days improvement percentage in severe patients after different treatment.

The primary and secondary outcomes are shown in Table 2 and Fig 1. As shown in Table 2, the 28-day composite outcomes of disease progression in total population were similar between Paxlovid plus GCS group and GCS group (12.1% *vs* 15.1%, *P* = 0.585). However, the 28-day composite outcomes of severe patients in Paxlovid plus GCS group was significantly lower than that in GCS group (16.9% *vs* 33.8%, *P* = 0.013), as shown in Fig 1 and Table 2.

**Table 2:** Outcomes of Omicron infected patients after different treatment.

	Paxlovid plus GCS (n = 99)	GCS (N = 167)	*P* value
**28-day composite outcome, n (%)**	12 (12.1%)	25 (15.1%)	0.585
Moderate	1 (2.9%)	1 (1.11%)	0.459
Severe	11 (16.9%)	24 (33.8%)	0.013
**Hospitalization time (days)**	11 (7, 16)	10 (6, 16)	0.104
Moderate	7 (5, 10)	9 (5.5, 13)	0.485
Severe	15 (9, 18)	17 (13, 19)	0.008
**Days of viral RNA clearance**	6 (3, 10)	8 (4, 15)	0.015
Moderate	5 (3, 6)	6 (5, 9)	0.128
Severe	8 (7, 10)	11 (9, 15)	0.001

The 28-day composite outcome was presented as n(%). The hospitalized time and viral RNA clearance days were presented as median and quartile.

Although the median hospitalized time were similar between two groups (11 days *vs* 10 days, *P* = 0.104), the hospitalized time of severe patients in Paxlovid plus GCS group was significantly lower than that in GCS group (15 days *vs* 17 days, *P* = 0.008). The viral RNA clearance time in Paxlovid plus GCS group was also significantly faster than that in GCS group (6 days *vs* 8 days, *P* = 0.015), especially in severe patients (8 days *vs* 11 days, *P* = 0.0101).

### Independent risk factors Cox regression analysis of subgroups

Next we evaluated the risk of composite disease progression outcome by subgroups of selected baseline characteristics. As shown in Table 3, Cox analysis showed the benefit of Paxlovid plus GCS treatment in sub-group of multiple organs dysfunction syndrome (MODS) (OR: 9.21, 95%CI: 1.962–43.148, *P* = 0.005), CRP ≥ 36 mg/L (OR: 5.74, 95%CI: 1.15–28.6, *P* = 0.029), d-dimer ≥ 1 µg/L (OR: 5.25, 95%CI: 1.05–26.19, *P* = 0.037) and creatinine ≥ 90 µmol/L (OR: 4.86, 95%CI: 1.36, 17.35, *P* = 0.019).

**Table 3:** Cox analysis of the prognosis of patients with Omicron treated with Paxlovid plus glucocorticoids.

Variables	OR(95%CI)	P value
Female	0.53(0.15, 1.87)	0.381
Age (≤70 years)	5.42 (0.65, 45.18)	0.159
MODS	9.21 (1.96, 43.15)	0.005
Leukocytes (≥7.0 × 10^9^/L)	1.76 (0.56, 5.56)	0.390
Lymphocyte(≥0.8 × 10^9^/L)	0.13 (0.03, 1.51)	0.195
CRP (≥36 mg/L)	5.74 (1.15, 28.63)	0.029
D-Dimer (≥1.0 ug/mL)	5.25 (1.05, 26.19)	0.037
Troponin, (≥20 pg/mL)	3.14 (0.79, 12.43)	0.111
NT-proBNP (≥300 pg/mL)	3.75 (0.73, 19.39)	0.173
Creatinine (≥90 µmol/L)	4.86 (1.36, 17.35)	0.019

## Discussion

In this multiple-centers study, we evaluated the efficacy of Paxlovid plus glucocorticoid in Omicron patients with hypoxaemia. Our results indicated that Paxlovid plus glucocorticoid significantly improved the 28-day composite outcome of Omicron patients with hypoxaemia. As far as we known, it is the first study directly providing the evidence about combination of Paxlovid plus glucocorticoid in severe Omicron patients or Omicron subjects with hypoxaemia. Our study, at least partly, support the use of Paxlovid in severe COVID-19 patients.

Update, Omicron variant had lower destruction ability compared with previous variants. However, the severe and critical disease still carry a high risk of worsened prognosis, especially in elders and subjects with multiple underlying disorders [20–22]. In COVID-19 patients with hypoxaemia, glucocorticoids have been proved reducing the hazard of death and decreased ventilator dependence [7–10]. Glucocorticoids may inhibit cytokines storm and modulate inflammation-mediated lung injury, reducing progression to respiratory failure and death [23,24]. There are two kinds of glucocorticoids in our study: dexamethasone and methylprednisolone. Dexamethasone was the primary glucocorticoids used in RECOVERY trials and other studies, with more clinical evidence [8–10]. Methylprednisolone is a more common used drugs in pulmonary disorders. But, it is unclear whether methylprednisolone is superior to dexamethasone in severe COVID-19 patients or not. Early studies, such as RECOVERY trial or other trials, selected dexamethasone for its advantage of cheap, feasibility and long-acting anti-inflammation [8–10]. In recent studies methylprednisolone was used and it achieved similar efficacy to dexamethasone [25,26]. Some studies indicated the superior efficacy of methylprednisolone [10,27]. Up to date, which types of glucocorticoids is more suitable for different sub-group subjects need further evaluation.

Another dispute is the different effect of glucocorticoids on viral clearance. Some reports and multivariate Cox regression analysis revealed that systemic glucocorticoid administration was the independent factor associated with delay of nucleic acid negative conversion time [28,29]. Other studies identified that SARS-CoV-2 clearance was not affected by systematic glucocorticoids use [30,31]. There is evidence that high doses of glucocorticoids prolonged the clearance of SARS-CoV-2, rather than low doses [32]. So, the suitable dose and duration of systematic glucocorticoids administration need further examination.

Theoretically, Paxlovid administration has the advantage of shortening the viral RNA shedding duration, which could ameliorate virus-associated dysfunction and cytokines storm in severe patients. However, most of the clinical evidences about Paxlovid came from trials in mild to moderate COVID-19 patients. A recent large scale open-label, multicenter, randomized controlled trial demonstrated that Paxlovid did not reduce the risk of all-cause mortality on day 28 and the duration of SARS-CoV-2 RNA clearance in hospitalized adult severe COVID-19 patients with comorbidities [33]. There is a lack of direct evidence about Paxlovid administration in Omicron subjects with hypoxaemia or in severe stage. As far as we know, our study provided the first evidence that the use of Paxlovid in severe Omicron patients or those with hypoxemia improves prognosis by reducing viral clearance in these subjects.

Our study revealed that the combination of antiviral drugs Paxlovid with glucocorticoids inhibited disease progression in Omicron patients. These results are consistent with some previous reports which focused on the efficacy of antiviral drugs remdesivir plus glucocorticoids in COVID-19 patients [17,34,35]. Several reports demonstrated that remdesivir plus dexamethasone treatment is associated with significant reduction in mortality, length of hospitalization, and faster SARS-CoV-2 clearance, compared to dexamethasone alone [17,34,35]. However, other reports identified that combination of remdesivir with dexamethasone did not bring any additional benefits [18,19]. These contrary results might result from several reasons. First, the baseline characteristics of COVID-19 patients in these studies were different, including SARS-CoV2 variant, ages, race and comorbidity. Second, the drugs administration time points varied in different studies. It might result in giant difference when remdesivir was administrated before, after or simultaneously with glucocorticoids. Third, the dose (from 6 mg/d to 20 mg/d) and duration (from 7 to14 days) of glucocorticoids in these varied markedly. Based on these, a large scale randomized controlled trial could provide more convincing evidences.

There are some limitations in our study. Firstly, it is uncertain whether other antiviral drugs (molnupiravir and Azvudine) both benefited to Omicron patients with hypoxaemia or not. Secondly, which kind of glucocorticoids (dexamethasone or methylprednisolone) was superior in severe COVID-19 patients? As the limited numbers of enrolled subjects, our study cannot answer this question. Thirdly, our study is only a prospective observing study with limited number of subjects. Maybe a matching methods for group comparisons and random controlled trials with larger scale would provide more persuasive evidence.

Conclusively, this multiple-centers prospective study demonstrated that the combination of Paxlovid with glucocorticoids was conducive to the 28-day composite outcome in Omicron patients with hypoxaemia. It might provide a few evidences about the treatment strategy of antiviral plus glucocorticoids in COVID-19 patients with hypoxaemia. Large-scale randomized controlled trials are need to confirm this conclusion.

## Supporting information

S1 Renamed_58beb. (JPG)

S2 Renamed_208b2. (PZFX)

S3 Renamed_e17b7. (XLSX)
