# Supplementary figures and images for: Paxlovid plus glucocorticoids treatment in severe Omicron infected patients with hypoxaemia: A prospective multiple-center cohort study
Source: PLoS One. 2025 Sep 26;20(9):e0328929. doi: 10.1371/journal.pone.0328929 (PMC12469179; doi:10.1371/journal.pone.0328929)

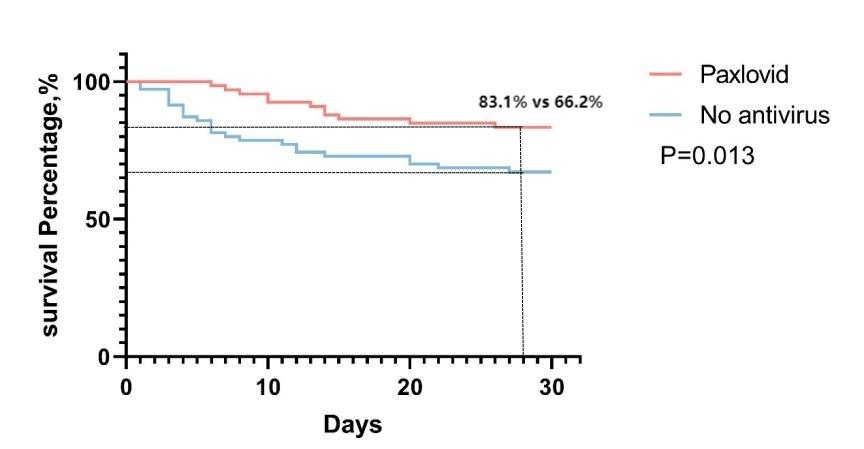

Supplement: S1 — (JPG) [file pone.0328929.s001.jpg]
